# The impact of K-Ras Gly12 mutants on homeostasis and tumorigenesis in the colonic epithelium

**DOI:** 10.1038/s41388-026-03771-3

**Published:** 2026-04-10

**Authors:** Moon Hee Yang, Shikha Sheth, Bing Shui, Isabella N. Grabski, Samuel Seongmin Park, Ana Cobo, Rafael Irizarri, John V. Heymach, Lukas E. Dow, Kevin M. Haigis

**Affiliations:** 1 Department of Cancer Biology, Dana-Farber Cancer Institute, Boston, MA USA; 2 Department of Medicine, Brigham & Women’s Hospital, Harvard Medical School, Boston, MA USA; 3 Department of Data Science, Dana-Farber Cancer Institute, Boston, MA USA; 4 Department of Biostatistics, Harvard T.H. Chan School of Public Health, Boston, MA USA; 5 Department of Thoracic/Head and Neck Medical Oncology, The University of Texas MD Anderson Cancer Center, Houston, TX USA; 6 Sandra and Edward Meyer Cancer Center, Weill Cornell Medicine, New York, NY USA; 7 Graduate School of Medical Sciences, Weill Cornell Medicine, New York, NY USA; 8 Department of Medicine, Weill Cornell Medicine, New York, NY USA

**Keywords:** Cancer genetics, Oncogenes

## Abstract

K-Ras represents one of the most frequently mutated and therapeutically relevant oncogenic drivers. Clinical and epidemiological data suggest association between specific KRAS alleles, therapeutic responses, and patient outcomes, however, direct experimental validation of these relationships has been limited. In this study, we investigate the differential impacts of three most common K-Ras G12 mutants (K-Ras^G12C^, K-Ras^G12D^, and K-Ras^G12V^) in the colon using in vivo models. Although tumors harboring different Gly12 mutants exhibited no obvious histological differences, their effects on survival outcomes and therapeutic responses displayed pronounced allele specific differences. Transcriptomic analyses revealed an allele-agnostic signature enriched for gene sets associated with MAPK signaling, receptor tyrosine kinase pathways, and immune-modulating pathways, relative to K-Ras WT tumors. In contrast, the allele-specific signature revealed marked enrichment of Notch and Wnt/β-catenin signaling pathways in K-Ras^G12C^ tumors. Pharmacological inhibition of these pathways, in combination with a K-Ras^G12C^ inhibitor, led to either addictive or synergistic reduction in tumor cell viability, in an allele-specific manner. These findings highlight the distinct biological consequences of individual K-Ras G12 mutations in colonic tumorigenesis and underscore therapeutic relevance of allele-specific signaling dependencies, offering a foundation for the development of effective, allele-informed therapeutic strategies for K-Ras mutant cancers.

## Introduction

K-Ras is a small GTPase that functions as a molecular switch, regulating signaling pathways vital for cell growth, differentiation, and survival. Its activity depends on its nucleotide binding state, cycling between an active GTP-bound form and an inactive GDP-bound form. Mutations in the *KRAS* gene are highly prevalent in some of the most aggressive cancers, including non-small cell lung cancer (NSCLC), colorectal cancer (CRC), and pancreatic ductal adenocarcinoma (PDAC) [1, 2]. The majority of these activating missense mutations occur at Gly12, leading to impaired GTP hydrolysis and sustained activation of K-Ras signaling.

Across cancers, the spectrum of K-Ras mutations exhibits considerable heterogeneity, including which amino acid is mutated and the specific mutations at a given residue. For instance, G12C mutations account for nearly 50% of all Gly12 mutations in NSCLC, however, these mutations are rare in PDAC, where mutations such as K-Ras^G12V^ and K-Ras^G12D^ predominate. Beyond the variation in allele frequency, these mutations differentially influence the biochemical properties of K-Ras. While all Gly12 mutants exhibit reduced GAP-mediated hydrolysis, some mutants, such as K-Ras^G12C^, retain intrinsic hydrolysis activity [3]. Interestingly, a recent study indicates the K-Ras^G12C^ is sensitive to hydrolysis induced by a non-canonical GTPase-activating protein (GAP), RGS3 [4], suggesting that there is more to learn about the regulatory mechanisms of mutant forms of K-Ras. Moreover, while K-Ras^G12C^, K-Ras^G12D^, and K-Ras^G12V^ maintain intrinsic nucleotide exchange rates comparable to wild-type (WT), their ability to undergo SOS-mediated exchange is compromised, with K-Ras^G12C^ showing a particularly high resistance to SOS1-mediated exchange [5].

In addition to their distinct biochemical properties, Gly12 mutations display distinct biological behaviors in cancer. In NSCLC, *KRAS*^*G12D*^ mutation is associated with an inferior response to standard chemotherapy, whereas *KRAS*^*G12V*^ exhibits increased sensitivity to platinum-based therapy [6–8]. Notably, *KRAS*^*G12V*^ and *KRAS*^*G12C*^ mutations are associated with poor survival of NSCLC patients and poor prognosis in CRC patients, while *KRAS*^*G12D*^ exhibit variable prognostic implications [9–14]. Interestingly, these three Gly12 mutations demonstrate distinct co-mutation networks that are cancer type-specific. These findings underscore the importance of understanding allele-specific downstream signaling engagement to inform more precise therapeutic strategies.

In this study, we systematically compared the three predominant K-Ras Gly12 mutant alleles – K-Ras^G12C^, K-Ras^G12D^, and K-Ras^G12V^ – in the context of the mouse colonic epithelium to delineate both shared and allele-specific signaling modulation underlying their differential tumor phenotypes. These data elucidate the mechanisms driving allelic selection and provide insights for developing efficient targeted therapies for K-Ras mutant colon cancers.

## Materials and methods

### Animal studies

All animal studies were approved by the Institutional Care and Use Committee (IACUC) at Beth Israel Deaconess Medical Center. Mice were fed *ad libitum* and housed in a barrier facility with a temperature-controlled environment and twelve-hour light/dark cycle. *Fabp1-Cre* (Strain:01XD8), *Apc*^*2lox14*^ (Strain:01XP3), and *Kras*^*LSL-G12D*^ (Strain:01XJ6) mice were obtained from the NCI Mouse Repository. *Kras*^*LSL-G12C*^ mice were generated as previously described [15]. *Kras*^*LSL-G12V*^ mice were kindly provided by David Tuveson (Cold Spring Harbor Laboratory). Experimental mice were maintained on a genetic background that was 80 to 95% C57BL/6, and genetic background was confirmed by SNP genotyping (Jackson Laboratory, Bar Harbor, ME, USA). Survival curves for tumor-bearing mice were analyzed using log-rank (Mantel-Cox) test.

### Tissue staining, IHC, and IF

Tissues were harvested from mice, fixed in 10% formalin overnight at room temperature, and processed into paraffin blocks. Tissue sections (5 μm) were deparaffinized using a standard xylene and ethanol series. Hematoxylin and eosin staining protocols were performed following standard protocols. For crypt height analyses, crypts were measured using analysis software associated with the VS120 slide scanner (Olympus, Tokyo, Japan) as described previously [16].

For IHC, deparaffinized sections were subjected to antigen retrieval in a pressure cooker in Target Retrieval Solution, pH 6 (DAKO, Glostrup, Denmark). IHC was performed with the EnVision+ HRP kit (DAKO). Primary antibodies were diluted in Antibody Diluent (DAKO) and incubated overnight at 4 °C. Images were acquired using an Olympus VS120 slide scanner. For IF, following antigen retrieval, sections were incubated in Protein Block, Serum-Free (DAKO) for 20 min at room temperature. Primary antibodies were diluted in Antibody Diluent (DAKO) and incubated overnight at 4 °C. Slides were then washed in PBS, followed by incubation with secondary antibodies diluted in Antibody Diluent for 1 h at room temperature in the dark. After incubation, slides were stained with DAPI (BD Bioscience, San Jose, CA, USA) for an hour at room temperature in the dark. Coverslips were mounted using ProLong Diamond (Invitrogen, Carlsbad, CA, USA).

For EdU (5-ethynyl 2’-deoxyuridine) staining, the Clik-iT EdU reaction was performed according to the manufacturer’s instructions. Briefly, deparaffinized slides were incubated in 0.5% Triton X-100 solution for 20 min, followed by incubation in EdU reaction cocktail for 30 min at room temperature in the dark. After washing with PBS, slides were incubated with DAPI and mounted with coverslip using ProLong Diamond. All immunofluorescence images were acquired using an VS120 Slide Scanner (Olympus). Primary antibodies included anti-Cytokeratin 20 (CST, Cat#13603, Danvers, MA, USA), anti-E-cadherin (BD Bioscience Cat#610181), anti-lysozyme (Thermo Fisher Scientific, Cat#RB-372-A1, Waltham, MA, USA.) and anti-Mcm6 (Abcam, Cat#ab201683, Cambridge, UK). Fluorescent secondary antibodies included anti-mouse IgG2a Alexa Fluor 594 (Thermo Fisher Scientific, Cat#A-21135) and anti-rabbit Alexa Fluor 647 (Thermo Fisher Scientific, Cat#A-31573).

### Ras-GTP pull down and western blotting

Glutathione S-transferase protein fused Raf binding domain (GST-RBD) conjugate beads were produced as described [17]. Frozen tissues were lysed in MLB lysis buffer [25 mM HEPES, 150 mM NaCl, 1% NP-40, 0.25% Sodium Deoxycholate, 10% glycerol and 10 mM MgCl_2_] supplemented with a protease inhibitor cocktail (Roche, Basal, Switzerland) and phosphatase inhibitor cocktails (Sigma, St. Louis, MO, USA). Equal amounts of protein lysates were incubated with 15μl of GST-RBD beads. The precipitated proteins were resolved on an SDS-PAGE gel, and immunoblotting was performed to detect GTP-bound Ras proteins using anti-Ras10 antibody (Millipore, Cat#05-516, Burlington, MA, USA).

To analyze Ras downstream signaling components, immunoblotting was performed according to standard protocols. Immunoblot images were quantified using Image Studio Software (LI-COR^®^, Lincoln, NE, USA, RRID:SCR_015795). Primary antibody included: anti-Ras10 (Millipore Cat#05-516, RRID:AB_11211664), anti-KRAS (Proteintech, Cat#12063-1-AP, Rosemont, IL, USA), anti-Akt (CST Cat#2920), anti-phospho-Akt (Ser473; CST Cat#4060), anti-Erk1/2 (CST Cat#4696), anti-phospho-Erk1/2 (CST Cat#4377), anti-Mek1/2 (CST Cat#4694), anti-phospho-Mek1/2 (CST Cat#9154), anti-β-catenin (CST Cat#9562), anti-GAPDH (CST Cat#5174), and anti-Vinculin (Sigma, Cat#V9131). Secondary antibodies included: anti-mouse IgG Alexa Fluor 680 (Thermo Fisher Scientific, Cat#A21058) and anti-rabbit IgG Alexa Fluor 800 (Thermo Fisher Scientific, Cat#A32735).

### Reverse phase protein array (RPPA) analysis

Tissues were lysed in RPPA lysis buffer [1% Triton X-100, 50 mM HEPES, pH 7.4, 150 mM NaCl, 1.5 mM MgCl_2_, 1 mM EGTA, 100 mM NaF, 10 mM Na pyrophosphate, 1 mM Na_3_VO_4_, and 10% glycerol], supplemented with a protease inhibitor (Roche) and a phosphatase inhibitor (Roche). Protein lysates were quantified using a bicinchoninic acid assay (Pierce, Waltham, MA, USA), diluted to a concentration of 1.5 mg/mL using lysis buffer, and mixed with 4X SDS buffer [40% glycerol, 8% SDS, 0.25 M Tris-HCl, pH 6.8, 10% 2-mercaptoethanol]. Lysates were serially diluted and arrayed on nitrocellulose-coated slides (Grace Bio Lab, Bend, OR, USA) using Quanterix Micro Arrays system (Quanterix, Model#2470, Billerica, MA, USA), with standard lysates and control spots included. Protein array was probed with a validated primary antibody and incubation with a biotin-conjugated secondary antibody. Signals were amplified using DAKO Cytomation-Catalyzed System (DAKO) and visualized by DAB colorimetric reaction. RPPA images were analyzed with customized software to assess the intensity. Antibodies showing a dominant band on western blotting and a Pearson correlation coefficient greater than 0.7 with RPPA were selected. Each dilution curve was fitted with a logistic model (“Supercurve Fitting” developed by the Department of Bioinformatics and Computational Biology in MD Anderson Cancer Center, http://bioinformatics.mdanderson.org/OOMPA). Protein concentrations of each set of slides were then normalized for protein loading. Log_2_-normalized and de-batched data were used to compare protein levels across genotypes of a given tissue. One-Way ANOVA with Tukey’s Multiple Hypothesis Test was used to determine significant differences between genotypes for each protein.

### Bulk RNA-seq analysis

Total RNA was isolated using the RNeasy Plus Universal Kit (Qiagen, Hilden, Germany), and messenger RNA was enriched using Poly-A enrichment. RNA libraries were prepared using NEBNext Ultra II Directional RNA Library Prep Kit for Illumina (NEB, Ipswich, MA, USA) according to the manufacturer’s instructions. Libraries were sequenced on Nextseq500 sequencer (Illumina, San Diego, CA, USA) with 10 million reads per sample in 75 bp single-read mode. Salmon (1.4.0) pseudo-aligner was used for sequencing alignment, with the Ensembl mm10 genome (Mus_musculus.GRCm38) as reference. Differential gene expression analysis was done using “DESeq2” (1.28.0) with default settings. Pre-ranked GSEA analysis was performed with the GSEA software v4.2.3 with MsigDB Hallmark gene-set, ranked by gene expression LFC.

### Small molecule inhibitor treatments in organoids

Mouse colonic epithelial organoids were derived from colons harvested from *Fabp1-Cre; Kras*^*LSL-mutant/+*^ mice and maintained as previously described [18]. Tumor organoids were derived from tumors harvested from *Fabp1-Cre; Apc*^*2lox14/+*^*; Kras*^*LSL-mutant/+*^ mice with different *Kras* genotypes (*Kras WT*, *Kras*^*G12C*^, *Kras*^*G12D*^, or *Kras*^*G12V*^) and maintained as described previously [19]. For inhibitor treatment, organoids were plated in 384 well at a concentration of 8×10^2^ cells per well. 24 h post-plating, compounds were added to each well in a 12-point dose curves along with DMSO controls using a D300e digital drug printer (Tecan, Mannedorf, Switzerland). Cell viability was assessed on day 6 post treatment using CellTiter Glo 3D (Promega, Madison, WI, USA), according to the manufacturer’s instructions.

## Results

### Effects of K-Ras Gly12 mutations on intestinal homeostasis

Expression of K-Ras^G12D^ dysregulates intestinal homeostasis by promoting epithelial hyperplasia and disrupting secretory cell differentiation via MAPK signaling pathway activation [20, 21]. In comparison, alleles with weaker biochemical activity, such as K-Ras^A146T^ and K-Ras^G13D^, elicit more modest phenotypic changes in the intestinal epithelium [16, 22]. Given the well-established impact of oncogenic K-Ras on intestinal homeostasis, we first sought to assess the effect of K-Ras Gly12 mutants in this physiological context. To achieve this, we crossed mice carrying conditional activating alleles of K-Ras (*K-Ras*^*LSL-G12C*^, *K-Ras*^*LSL-G12D*^, and *K-Ras*^*LSL-G12V*^) to *Fabp1-Cre* mice, which express Cre recombinase in the distal small intestinal and colonic epithelia [23].

**Fig. 1:**
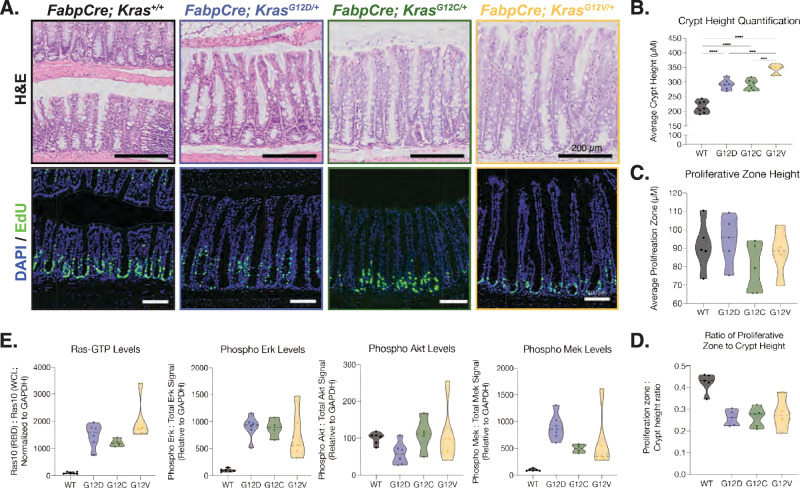
The K-Ras Gly12 mutants have similar impact on the colonic epithelium. **A** Histology of colons expressing different K-Ras mutants. The top row shows representative H&E images of colonic epithelium from 8-week-old mice carrying the indicated genotype. The bottom row displays representative immunofluorescence images highlighting proliferating cells, labeled with EdU (5-ethynyl 2’-deoxyuridine). **B** Quantification of crypt height in colonic epithelium. All *Kras* Gly12 mutants induce hyperplasia in colonic epithelium. All animals carried the *Fabp1-Cre* transgene and various *Kras* alleles. Crypt heights were measured every 5 crypts along the length of the colon, and the average crypt heights were calculated from a random sample of 100 measurements. *N* = 7 for K-Ras WT and *N* = 6 for K-Ras^G12D^, K-Ras^G12C^ and K-Ras^G12V^. *** *P* < 0.001, **** *P* < 0.0001, One-way ANOVA (Tukey’s multiple comparison test). **C** Quantification of average proliferative zone height for indicated genotypes. The height of the EdU-positive portion in the crypt was measured along the colon of indicated *Kras* genotypes. *N* = 5 for K-Ras WT, K-Ras^G12D^ and K-Ras^G12C^, and *N* = 4 for K-Ras^G12V^. **D** The ratio of proliferative zone to total crypt height. The ratio was calculated from each measurement and averaged across all measurements within each colon. N = 5 for K-Ras WT, K-Ras^G12D^ and K-Ras^G12C^, and *N* = 4 for K-Ras^G12V^. **E** Activation of Ras and its canonical downstream signaling pathways in colons expressing different K-Ras mutants. The levels of Ras-GTP were assessed by affinity precipitation of Ras-GTP using Raf-RBD pull-down. The level of K-Ras activity and MAPK signaling activation is increased in colons expressing K-Ras Gly12 mutants, while PI3K signaling activation remains unaffected. MAPK signaling activation was measured by western blotting for phosphorylation of Mek at Ser217/221 and Erk at Thr202/204. PI3K signaling activation was determined by phosphorylation of Akt at Ser 473. For each sample, quantification of total protein signal was normalized to Gapdh, and the phospho-protein signal was then normalized to this value. N = 6 for K-Ras WT, K-Ras^G12D^ and K-Ras^G12V^, *N* = 4 for K-Ras^G12C^ in two independent experiments.

In agreement with previous findings [15, 20], expression of all three K-Ras Gly12 mutants in the colonic epithelium induced marked hyperplasia, characterized by increased crypt height and goblet cell hypertrophy, with the most pronounced changes observed in mice expressing K-Ras^G12V^ (Fig. 1A, B). Despite their profound effect on crypt height, none of the alleles significantly expanded the proliferative zone, as assessed the height of EdU labeled region (Figs. 1A, C). Although minor variation in EdU staining intensity was observed among individual samples, quantitative analysis revealed comparable proliferative zone heights across *Kras* genotypes (Fig. 1C). Notably, the ratio of the proliferative zone to total crypt height was decreased in colonic epithelium expressing mutant K-Ras compared to WT (Fig. 1D), suggesting that increased crypt height is, at least in part, due to the accumulation of differentiated cells. These findings are consistent with previous observations [15] and support a role for K-Ras Gly12 mutations in disrupting the dynamics of intestinal epithelial cell turnover.

In addition to hyperplasia, we assessed the impact of K-Ras Gly12 mutations on secretory cell lineage differentiation using immunohistochemistry for lysozyme, a marker of Paneth cells, in the ileum. Paneth cells were completely depleted in ileum expressing any of the K-Ras Gly12 mutants (Fig. S1A). Consistent with their phenotypic impact on intestinal homeostasis, the levels of GTP-bound K-Ras and MAPK pathway activation were significantly elevated in colonic epithelia of these mice (Fig. 1E and Fig. S1B). Together, these results demonstrate that all three mutant alleles elicit comparable phenotypic alterations in intestinal epithelial homeostasis.

### Mutant-specific consequences of K-Ras Gly12 mutations in colon tumors

Although the three K-Ras Gly12 mutants exhibited comparable effects on intestinal homeostasis, multiple studies have highlighted their varying allele frequencies across different cancer types, as well as their therapeutic response to therapies [11, 24–26]. Considering these clinical and epidemiological observations, we aimed to explore the impact of Gly12 mutants in the context of colonic tumorigenesis. While oncogenic K-Ras activation is sufficient to perturb intestinal homeostasis, it is insufficient to drive tumorigenesis on its own and requires cooperation with loss-of-function mutations in tumor suppressor genes [20]. To model this, we crossed *Fabp1-Cre* mice harboring individual conditional *K-Ras* Gly12 alleles to those carrying a Cre-dependent allele of the Adenomatous polyposis coli (*Apc*) tumor suppressor gene [27].

**Fig. 2:**
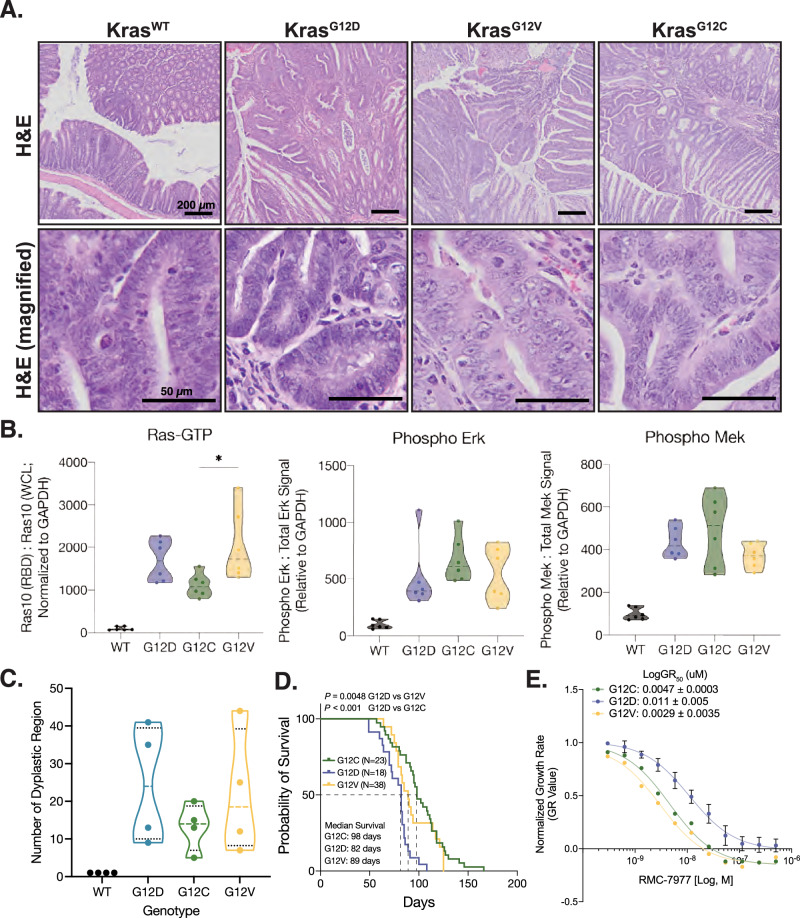
The impact of K-Ras Gly12 mutants in colonic tumors. **A** Histological analysis of the mouse colon tumors. Representative H&E images for mouse colon tumors carrying the given genotypes were used to assess tumor grade. No clear differences were observed between tumors with indicated genotypes. **B** Activation of K-Ras and MAPK signaling pathway in colon tumors expressing different K-Ras mutants. Left graph illustrates the quantification of Ras activity determined by accessing the level of GTP-bound Ras using the Raf-RBD pull-down assay. Ras activity is increased in the tumors expressing K-Ras Gly12 mutant alleles compared to those expressing Kras wild type, although tumors expressing Kras^G12C^ shows a modest increase compared to other Kras Gly12 mutants. Graphs in the middle and right display MAPK signaling activation in colon tumors expressing different Kras alleles determined by assessing Mek and Erk phosphorylation using western blotting. Both Mek and Erk phosphorylation are elevated in colon tumors expressing of K-Ras Gly12 mutants. For each sample, the quantification of total protein signal was normalized to GAPDH, and the phosphor-protein signal was then normalized to this value. *N* = 6 for all genotypes in two independent experiments. *P < 0.05, Mann-Whitney test. **C** Scatter plot demonstrating the number of tumors in *Apc*-mutant animals carrying the indicated genotypes. Each dot represents individual mouse bearing colon tumors. **D** Survival curves for colon tumor bearing mice with indicated genotypes. Mice expressing K-Ras^G12D^ exhibit significantly worse survival compared to those carrying other K-Ras Gly12 mutants. Survival differences between genotypes were analyzed using the Log-Rank (Mantel-Cox) test. Median survival for each group is shown in the graph. **E** Response of colon tumor organoids carrying different *Kras* Gly12 mutant alleles to pan-K-Ras inhibitor, RMC-7977, which targets GTP-bound K-Ras. Tumor organoids carrying different K-Ras Gly12 mutants exhibit differential responses to RMC-7977. In this study, three independent clones were used for K-Ras^G12D^ and K-Ras^G12C^, and one clone for K-Ras^G12V^. Each dot in the inhibitor response curve represents the average of triplicates of each clone from same genotype across two independent experiments, with curve fitting using nonlinear regression.

Colon tumors developing from all three K-Ras mutant genotypes displayed similar histological features, characterized as adenomas with low-grade dysplasia (Fig. 2A). As expected, the level of GTP-bound K-Ras was markedly elevated in tumors expressing mutant K-Ras compared to those with WT K-Ras. Notably, K-Ras^G12C^ tumors exhibited significantly lower levels of GTP-bound K-Ras compared to K-Ras^G12D^ and K-Ras^G12V^ tumors. Despite this fact, MAPK pathway activation, as assessed by downstream signaling readouts, was elevated to a similar extent in colon tumors expressing all three K-Ras Gly12 mutants (Fig. 2B and Fig. S2A). Interestingly, mice harboring K-Ras^G12C^ mutations developed fewer tumors than those with K-Ras^G12D^ or K-Ras^G12V^ mutations, which displayed comparable tumor numbers (Fig. 2C). Consistent with the tumor burden data, K-Ras^G12D^ tumor-bearing mice had the shortest overall survival, followed by K-Ras^G12V^ and K-Ras^G12C^ (Fig. 2D).

To further investigate how K-Ras Gly12 mutations influence cellular signaling dependencies, we evaluated the response of colon tumor organoids expressing different K-Ras Gly12 mutants to inhibitors targeting K-Ras. In these organoid models, K-Ras Gly12 mutants exhibited distinct sensitivities to RMC-7977, a tool compound to daraxonrasib (RMC-6236), a molecular glue that selectively targets the GTP-bound “ON” state [28, 29], and BI-2865, an allosteric inhibitor that stabilizes the GDP-bound “OFF” state [30]. Notably, K-Ras^G12D^ organoids were the most resistant to both inhibitors, whereas K-Ras^G12V^ organoids were the most sensitive (Fig. 2E and Fig. S2B). Interestingly, K-Ras^G12C^ organoids displayed selective sensitivity to the RMC-7977 but showed limited responsiveness to BI-2865. This discrepancy is likely attributable to the culture conditions used in this experiment, which included epidermal growth factor (EGF). EGF stimulation may shift K-Ras^G12C^ toward the GTP-bound state by activating the EGFR-SOS1-RAS exchange axis, thereby decreasing the intracellular GDP fraction available for OFF-state inhibitor engagement. Collectively, these findings suggest that while all Gly12 mutants display similar histological features and levels of MAPK pathway activation, their distinct phenotypic outcomes including differences in survival and therapeutic responses shed light on the need to further elucidate the allele specific molecular mechanisms driven by K-Ras Gly12 mutants in colon tumors.

### Mutant-agonistic transcriptional effects of K-Ras Gly12 mutants

**Fig. 3:**
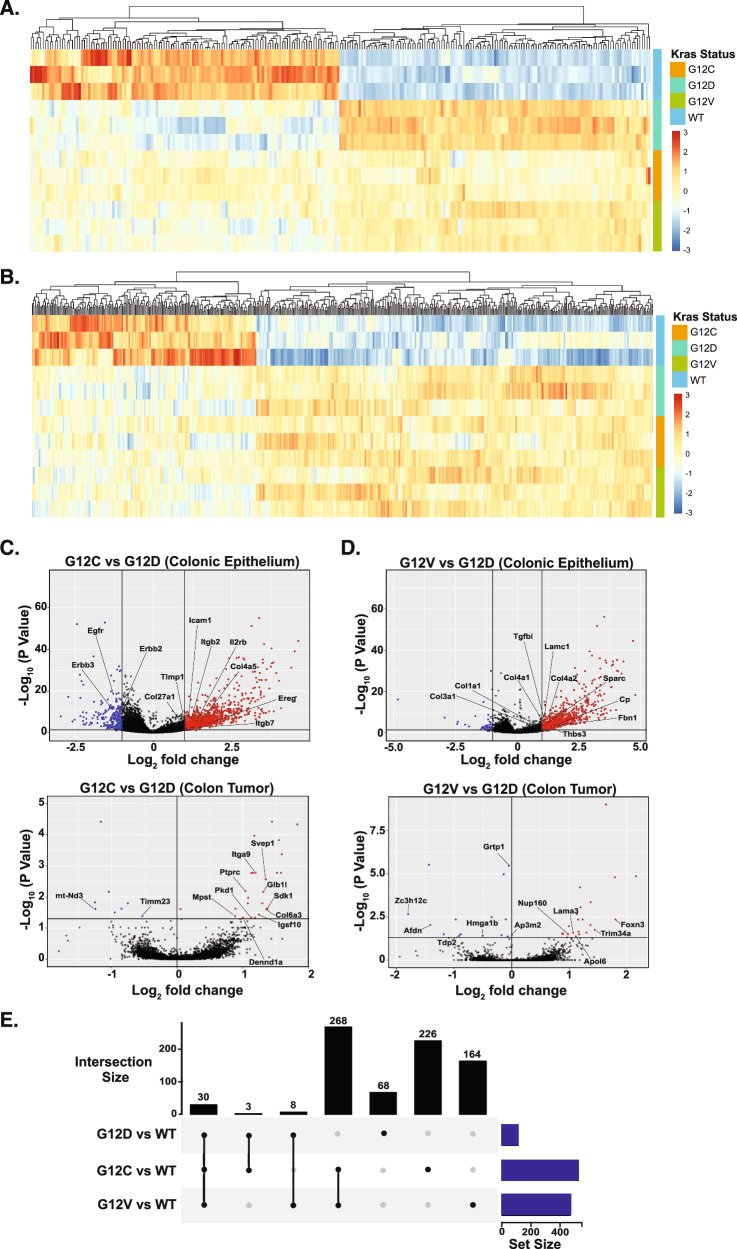
Impact of *Apc* mutation on the distinct gene expression regulated by K-Ras Gly12 mutants. **A** Heatmap displaying all DEGs in colonic epithelium from mice expressing the indicated K-Ras Gly12 mutant alleles compared with genetic controls. Each row represents the transcriptional expression in colonic epithelium carrying the indicated genotypes (*Fabp-Cre; Kras WT* or *Kras*^*G12C/D/V*^). *N* = 3 for each genotype. **B** Heatmap displaying all DEGs in colon tumors from mice expressing indicated K-Ras Gly12 mutant alleles compared with genetic controls. Each row represents the transcriptional expression in colon tumors carrying the indicated genotypes (*Fabp-Cre; Apc*^*2lox/+*^*; Kras WT* or *Kras*^*G12C/D/V*^). *N* = 3 for each genotype. **C, D** Volcano plots display DEGs in colonic epithelium or colon tumors expressing K-Ras^G12C^ **C** or K-Ras^G12V^ **D** compared to those expressing K-Ras^G12D^. The number of DEGs is markedly reduced in colon tumors compared to colonic epithelium. Genes labeled in the plots represent top-ranked DEGs based on both statistical significance and magnitude of fold change. **E** Upset plot illustrating the overlap of DEGs identified from each comparison between K-Ras Gly12 mutant alleles and K-Ras WT controls in colon tumors. Black bars represent the number of genes shared among or unique to specific *Kras* Gly12 mutant alleles (Intersection size), while blue bars indicate the total number of DEGs identified in each comparison (set size).

To gain further insight into the molecular impact of K-Ras Gly12 mutations, we performed transcriptomic profiling to examine gene expression changes in both colonic epithelium and colon tumors expressing different K-Ras mutants. As anticipated, the expression of mutant K-Ras induced substantial transcriptional alterations in both settings. Notably, in the colonic epithelium, K-Ras Gly12 mutants exhibited a gradient of transcriptional changes, with the most prominent changes observed in colonic epithelium expressing K-Ras^G12D^, followed by K-Ras^G12V^ and K-Ras^G12C^ (Fig. 3A). In line with this result, our gene set enrichment analysis (GSEA) demonstrated more extensive gene set-level changes in *K-Ras*^*G12D*^ tumors, followed by *K-Ras*^*G12V*^ and *K-Ras*^*G12C*^ tumors (Fig. S3A–C). However, this mutant-specific transcriptional gradient was not observed in colon tumors, where gene expression profiles appeared more similar across all three K-Ras Gly12 mutants (Fig. 3B).

Consistent with our hierarchical clustering, principal component analysis (PCA) revealed a clear separation in gene expression profiles among different K-Ras Gly12 mutants in the intestinal epithelium. Specifically, PC1 largely separated the samples based on genotype, with K-Ras^G12C^ clustering closer to WT, whereas K-Ras^G12D^ showed the greatest divergence from WT (Fig. 3C). In contrast, this clear separation was not observed in colon tumors, where gene expression profiles from all three K-Ras Gly12 mutants appeared more similar to each other and showed less distinction from K-Ras WT tumors (Fig. 3D). Furthermore, the number of differentially expressed genes (DEGs) in K-Ras^G12C^ or K-Ras^G12V^ tumors relative to K-Ras^G12D^ tumors were markedly reduced compared to the differences observed in colonic epithelium (Fig. 3E, F). DEG overlap analysis showed that K-Ras^G12C^ and K-Ras^G12V^ shared similar transcriptional profiles, whereas K-Ras^G12D^ appeared more distinct, although this divergence may partly reflect the smaller number of DEGs identified in this comparison. In addition, gene sets dysregulated by K-Ras Gly12 in colonic epithelium, including those associated with K-Ras signaling were also altered by *Apc* mutation, as observed in tumors with *K-Ras* WT compared with colonic epithelium harboring *K-Ras* WT (Fig. S3D). These overlapping effects on transcriptional regulation suggest that *Apc* mutation may override or mask mutant-specific transcriptional modulation induced by K-Ras mutations, thereby attenuating the distinct transcriptional impacts associated with individual *K-Ras* Gly12 alleles.

Despite the dampening effect of *Apc* mutation on the distinct transcriptional programs driven by individual K-Ras Gly12 alleles in colon tumors, the expression of mutant K-Ras still led to substantial transcriptional alterations compared to K-Ras WT tumors (Fig. 3B and 3D). Consistent with this observation, multiple clinical studies have reported that K-Ras Gly12 mutations are generally associated with worse outcomes relative to K-Ras WT tumors, although their impact relative to non-Gly12 K-Ras mutations (e.g., codon 13 mutations) remains variable and context dependent [31–33]. Motivated by these findings, we sought to define an allele-agnostic K-Ras Gly12 mutant transcriptional signature in our mouse model by comparing gene expression profiles of colon tumors harboring K-Ras Gly12 mutations with those expressing K-Ras WT.

**Fig. 4:**
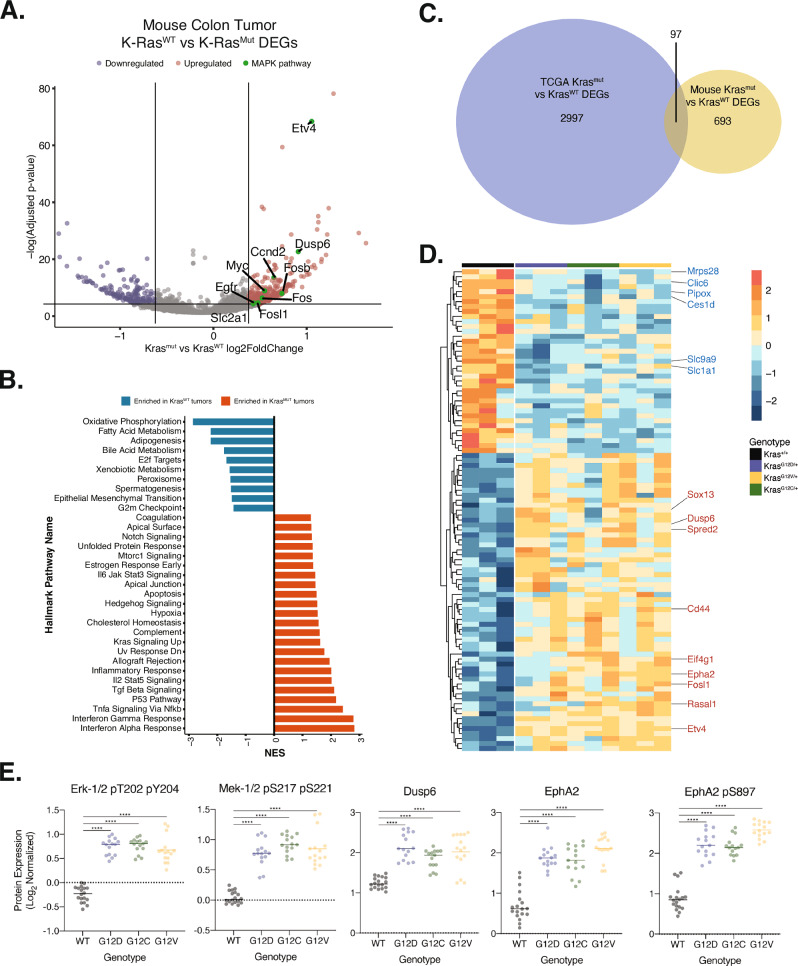
Mutant-agonistic effects of K-Ras Gly12 mutants in colon tumors. **A** Differentially expressed genes in K-Ras Gly12 mutant colon tumors compared to K-Ras WT tumors. Notably, MAPK signaling components (highlighted in green) involved in promoting cell proliferation are upregulated in K-Ras mutated tumors. LFC-cutoff = 0.5; adj-*p*-value < 0.05. **B** Gene Set Enrichment Analysis (GSEA) for dysregulated pathways specific to K-Ras mutated colon tumors. K-Ras mutant tumors are enriched for genes involved in the interferon response, TGF-β, TNF-α and p53 pathways, all of which are implicated in tumor progression. **C** Venn diagram displaying the overlap of differentially regulated genes in K-Ras Gly12 mutant human tumors and mouse tumors. The human colon tumor dataset was sourced from TCGA. The genes dysregulated in the same direction by all three K-Ras Gly12 mutants across both human and mouse datasets are included in Venn diagram. **D** Heatmap demonstrating the expression of overlapping genes (*n* = 97, from panel **C**) in mouse colon tumors. Genes highlighted in red are known to be regulated by MAPK hyperactivation and involved in K-Ras-driven tumorigenesis. **E** Allele-agonistic dysregulated proteins in K-Ras mutated mouse colon tumors. Dot plots of log normalized expression levels for proteins consistently dysregulated by all K-Ras Gly12 mutant alleles, relative to Kras *WT*. *p < 0.05; **p < 0.01; ***p < 0.001; ****p < 0.0001, One-way ANOVA (Tukey’s Multiple Comparison Test).

Our mutant-agnostic analysis revealed significant upregulation of key MAPK pathway targets involved in cell proliferation, including *Ccnd2, Fos, Fosb*, and *Myc*. Additionally, Dusp6, a negative feedback regulator of MAPK signaling, was upregulated, reflecting hyperactivation of the MAPK pathway. Increased expression of *Slc2a1*, encoding the GLUT1 glucose transporter, and *Egfr* was also observed, suggesting the presence of a positive feedback loop that may amplify MAPK signaling pathway (Fig. 4A). To further explore broader signaling alterations associated with K-Ras Gly12 mutations, we performed GSEA, which revealed upregulation of pathways involved in tumorigenesis and cancer progression, including TGF-β, NFκB, TNF-α, and p53 signaling (Fig. 4B). In contrast, pathways related to oxidative phosphorylation and other metabolic processes were downregulated, supporting the critical role of mutant KRAS in driving metabolic reprogramming in tumors (Fig. 4B).

To evaluate the clinical relevance of our findings, we next compared our mouse transcriptomic data with human colon cancer datasets from the TCGA COAD cohort [34]. Our cross-species analysis identified 97 genes that were dysregulated in the same direction by K-Ras Gly12 mutants in both mouse and human colon tumors (Fig. 4C). Many of these shared genes are involved in MAPK signaling activation and cancer cell proliferation, including *ETV4*, *FOSL1*, and *EPHA2* (Fig. 4D). Additionally, the expression of *RASAL1*, *DUSP6*, and *SPRED2*, all negative regulators of MAPK signaling, was up-regulated in K-Ras mutant tumors compared to K-Ras WT tumors in both species, further supporting the hyperactivation of MAPK signaling. Beyond MAPK signaling, we also observed upregulation of genes implicated in other pathways modulating tumor progression, including *EIF4G1* and *SOX13* [35–37].

Building on these transcriptomic observations, we examined proteomic changes using Reverse Phase Protein Array (RPPA) in colon tumors expressing different K-Ras mutants. Consistent with the transcriptomic findings, RPPA revealed elevated levels of phosphorylated Erk1/2, phosphorylated Mek1, and Dusp6 in tumors harboring K-Ras Gly12 mutants compared to those expressing K-Ras WT, confirming hyperactivation of the MAPK signaling pathway (Fig. 4E). Moreover, both expression and phosphorylation of Epha2 were increased across all three K-Ras Gly12 mutant tumors, which aligned with our transcriptomic profiling results (Fig. 4E). Taken together, these findings define a K-Ras Gly12 mutant associated signature that is conserved across both human and mouse colon tumors. The genes within this signature are linked to key tumor promoting pathways, including MAPK signaling, metabolic reprogramming, and feedback regulation.

### Mutant-specific therapeutic vulnerabilities in K-Ras Gly12 mutant colon tumors

**Fig. 5:**
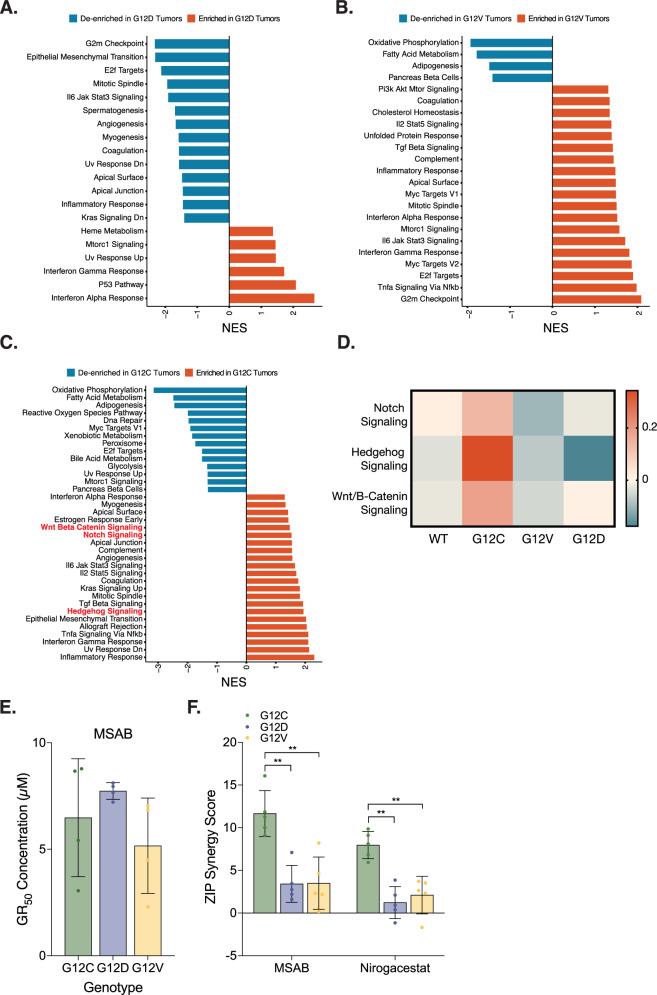
Mutant-specific effects of K-Ras Gly12 mutants in mouse colon tumors. **A–C** Hallmark gene sets significantly dysregulated in K-Ras^G12D^ (**A**) or K-Ras^G12V^ (**B**) or K-Ras^G12C^ (**C**) colon tumors, compared to those with K-Ras WT. Pathways highlighted in red are exclusively enriched in K-Ras^G12C^ mutant tumors. **D** Heatmap displaying GSVA normalized enrichment scores [32] for the indicated pathways in K-Ras WT, K-Ras^G12C^, K-Ras^G12D^, and K-Ras^G12V^ mouse colon tumors. **E** Response of K-Ras mutant mouse colon cancer organoids to β-catenin inhibition. The response to MSAB alone is not statistically different between organoids expressing different K-Ras Gly12 mutants. Plotted values indicate the concentrations at which 50% growth rate inhibition occurred in each genotype. Each dot represents an independent replicate. **F** ZIP synergy scores for combination treatments with MRTX849 in tumor organoids harboring K-Ras Gly12 mutations. Pharmacological Inhibition of Wnt/β-catenin or Notch signaling, when combined with KRAS^G12C^ specific inhibition, selectively abrogates the growth of K-Ras^G12C^ organoids compared with other genotypes. Synergy score was calculated using ZIP model, with each dot representing in an independent experiment. In all panels, error bar shows SD. **P < 0.01, Mann-Whitney test.

While all K-Ras Gly12 mutants modulate key oncogenic pathways such as TGF-β, NFκB, TNF-α, and p53 signaling, accumulating clinical evidence, together with our in vitro drug response data, indicates mutant-specific differences, particularly in therapeutic sensitivity (Fig. 2D and Fig. S2B) [11, 25, 26]. In light of these observations, we aimed to identify dysregulated pathways unique to each K-Ras Gly12 mutant, with the goal of uncovering potential allele-specific therapeutic vulnerabilities. To this end, we perform GSEA, comparing this gene expression profiles of tumors harboring each K-Ras mutation with those expressing K-Ras WT (Fig. 5A–C and Fig. S4A-B). Given the limited clinical efficacy of K-Ras^G12C^ inhibitors in colorectal cancer patients [38, 39], we focused on tumors harboring the K-Ras^G12C^ mutation. Notably, K-Ras^G12C^ tumors exhibited significant upregulated expression of gene sets associated with Notch, Hedgehog, and WNT/ β-catenin signaling pathways, while this was not observed in tumors driven by other K-Ras Gly12 mutants. This K-Ras^G12C^ specific transcriptional signature was further validated by Gene Set Variation Analysis (GSVA), which confirmed selective upregulated of these pathways exclusively in K-Ras^G12C^ tumors, in concordance with our GSEA results (Fig. 5D).

To explore the therapeutic relevance of these dysregulated signaling pathways, we evaluated the responses of tumor organoids expressing different K-Ras Gly12 mutants to inhibitors targeting these signaling pathways. Interestingly, organoids expressing all three K-Ras Gly12 mutants showed comparable response to MSAB, a direct inhibitor of β-catenin [40], indicating no inherent allele specific sensitivity to WNT inhibition (Fig. 5E). However, when MSAB was combined with MRTX849, a covalent K-Ras^G12C^ specific inhibitor, K-Ras^G12C^ organoids exhibited a dramatic and synergistic reduction in viability, an effect not observed in K-Ras^G12D^ organoids and with only modest, additive effects seen in K-Ras^G12V^ organoids (Fig. 5F and Fig. S5A). In addition, treatment with a γ-secretase inhibitor, Nirogacestat [41], alone resulted in limited efficacy across all three Kras Gly12 mutant organoids, with no clear allele-specific differences in response (data not shown). However, when combined with MRTX849, K-Ras^G12C^ organoids exhibited a pronounced cooperative effect in viability, suggesting that co-targeting K-Ras^G12C^ and Notch signaling pathways confers an additional therapeutic benefit (Fig. 5F and Fig. S4B).

To further examine the molecular basis of the allele-specific drug responses, we assessed canonical signaling activity in tumor organoids expressing K-Ras^G12C^, K-Ras^G12D^, or K-Ras^G12V^ after treatment with MRTX849 alone or in combination with MSAB or Nirogacestat. In K-Ras^G12C^ organoids, MRTX849 treatment markedly reduced Mek phosphorylation, whereas MSAB or Nirogacestat had no appreciable effect on MAPK activation (Fig. S6A), suggesting that Wnt or Notch signaling function in parallel to, rather than upstream of, the canonical RAS downstream signaling. This finding is consistent with their predominant roles in transcriptional regulation process rather than direct modulation of MAPK signaling. Interestingly, MSAB or Nirogacestat selectively suppressed downstream markers of Wnt or Notch signaling, respectively in K-Ras^G12C^ organoids, whereas these effects were minimal or absent in K-Ras^G12V^ or K-Ras^G12D^ organoids respectively (Fig. S6A–C), indicating relative resistance of these mutants to Wnt and Notch inhibition. Collectively, these findings suggest the therapeutic potential of rational combination strategies tailored to specific K-Ras mutants.

## Discussion

The oncogenic role of K-Ras signaling in cancer has been extensively characterized over the past several decades and direct therapeutic targeting of certain K-Ras mutants has recently become feasible. In conjunction with this therapeutic revolution, our understanding of context-specific oncogenic K-Ras signaling has greatly expanded over the last two decades. Indeed, the cellular consequences of K-Ras activation are shaped by various factors, including the specific K-Ras mutations [16, 22, 42], co-occurring genetic alterations [24], and tissue context [43]. Given the molecular and clinical heterogeneity of human cancers, delineating the functional roles of different K-Ras mutants across diverse biological contexts remains critical for refining precision strategies targeting K-Ras mutant cancers. In this study, we aimed to characterize the biological and molecular effects of the three most prevalent K-Ras Gly12 mutants in the context of the colon. Using genetically engineered mouse models with defined K-Ras mutations, we modeled early-stage colon tumorigenesis with a controlled genetic background, a crucial aspect not feasible with human tumors. Through transcriptomic and proteomic analyses, we discovered both the allele-agnostic signature common to K-Ras Gly12 mutants and allele-specific effects, most notably associated with K-Ras^G12C^ mutations in colon tumors.

Although tumors expressing different K-Ras Gly12 mutants exhibited similar histological features, the specific alleles had significant impacts on overall survival. Mice bearing K-Ras^G12D^ tumors exhibited the shortest survival, whereas those with K-Ras^G12C^ tumors demonstrated the most favorable survival outcomes, likely reflecting differences in tumor burden (Fig. 2). Notably, this mutant-specific survival phenotype differs from clinical observations in human colorectal cancers, where K-Ras^G12D^ mutants have not been consistently associated with inferior outcomes relative to other mutants [13, 14]. This discrepancy may reflect fundamental differences between human colorectal tumors and our GEMMs, with respect to genetic heterogeneity, tumor staging, and co-mutational landscape. In particular, the tumors in our mouse model more closely resemble low-grade adenomas, while human datasets are enriched for advanced stage, genetically complex tumors. Nevertheless, in line with these findings, tumor organoids expressing K-Ras^G12D^ exhibited the greatest resistance to direct K-Ras inhibition (Fig. 2). Given the high prevalence of K-Ras^G12D^ in human colon cancers and the early-stage nature of tumors in our mouse model, these results suggest that K-Ras^G12D^ mutant may confer signaling advantages that promote tumor progression and therapeutic resistance during the early phases of tumorigenesis.

In agreement with these findings, tumor organoids expressing K-Ras^G12D^ exhibited the greatest resistance to direct K-Ras inhibition by both “ON” and “OFF” state inhibitors (Fig. 2). This pronounced resistance likely reflects a signaling advantage during tumor progression and also biochemical properties that confer reduced therapeutic responses. The differential nucleotide cycling behaviors of these mutants appear to play a critical role in shaping their drug sensitivities. Compared to K-Ras^G12D^, K-Ras^G12V^ is more responsive to SOS1-mediated nucleotide exchange, promoting rapid GDP-GTP turnover and a dynamic cycling state that increases its accessibility to both inhibitors. By contrast, K-Ras^G12D^ exhibits attenuated SOS1 responsiveness and slower GAP mediated hydrolysis, favoring a persistently GTP-loaded conformation that limits the formation of the GDP-bound population required for effective OFF-state inhibitor engagement [3, 5]. In addition, the relative lower affinity K-Ras^G12D^ to RMC-7977 further contributes to its reduced sensitivity [29]. Notably, while K-Ras^G12C^ exhibits resistance to OFF-state inhibitor through EGFR pathway activation under growth factor-rich conditions, K-Ras^G12D^ displays an intrinsic form of resistance that is independent of upstream receptor input. These distinct biochemical and signaling properties likely underlie the enhanced tumor fitness and therapeutic resistance conferred by the K-Ras^G12D^ allele in colorectal tumorigenesis.

In our transcriptomic results, tumor harboring K-Ras Gly12 mutations exhibited up-regulated expression of gene sets related to immune modulation, including interferon alpha (IFN-α) and interferon gamma (IFN-γ) response, allograft rejection, and inflammatory response pathways (Fig. 5). This result contrasts with prior studies demonstrating that mutant K-Ras suppresses IFN response pathways to facilitate immune evasion [43, 44], which is consistent with our finding in the colonic epithelium (Fig. S3). Notably, other prior studies have highlighted the pro-tumorigenic roles of interferons, including their involvement in tumor progression and metastasis under certain contexts [45, 46]. Considering this dual role of interferon signaling and the early-stage nature of our tumor models, K-Ras Gly12 mutations may enhance pro-tumorigenic inflammatory signaling through interferon pathways, contributing to tumor progression in the early stages. Supporting this hypothesis, genes associated with IFN response pathways were highly enriched in K-Ras^G12D^ tumors compared to other K-Ras Gly12 alleles, aligning with the higher tumor burden and worse survival outcomes observed in these mice (Fig. 2). Such allele-dependent immune signatures imply that K-Ras mutations may not only alter tumor-intrinsic signaling, but also remodel the surrounding microenvironment through changes in cytokine networks and immune cell interactions, potentially influencing allele-specific tumor progression dynamics. However, the extent to which each K-Ras Gly12 mutant differentially shapes the tumor immune microenvironment in colon tumor remains unclear and warrants further investigation.

In addition to the effect on immune-modulating pathways, we also observed upregulated expression of genes associated with receptor tyrosine kinase (RTK) signaling, including *Egfr, Vegfr2* and *Epha2*, in K-Ras Gly12 mutant tumors based on both transcriptomic and proteomic analyses. Particularly, Epha2 was highly expressed and activated across in all three Gly12 mutant tumors relative to WT (Fig. 4), consistent with a previous report [47]. Given the established role of Epha2 in promoting resistance to tyrosine kinase inhibitors (TKIs), invasion, and tumor progression in colorectal cancers [47, 48], Epha2 may represent a promising therapeutic target for K-Ras Gly12 mutant cancers.

Beyond these mutant-agnostic changes, we also identified mutant specific transcriptional alterations. K-Ras^G12C^ tumors exhibited distinct transcriptional enrichment of gene sets linked to Wnt/β-catenin signaling, Notch signaling, and Hedgehog signaling (Fig. 5). Although inhibition of Notch or WNT pathways alone did not produce allele-specific effects in K-Ras^G12C^ organoids, combining these inhibitors with the K-RAS^G12C^ specific inhibitor MRTX849 revealed pronounced vulnerabilities. In addition to combinatorial effect of Notch/K-Ras inhibition, combination treatment with MSAB, a selective β-catenin inhibitor, produced a synergistic reduction in viability in K-Ras^G12C^ organoids, despite the absence of allele-specific effects with WNT inhibition alone (Fig. 5 and Fig. S6). Given the presence of *Apc* mutations and baseline WNT activation across all tumors, the further transcriptional upregulation of WNT target genes specifically in K-Ras^G12C^ tumors suggests that K-Ras^G12C^ specific signaling properties, potentially involving non-canonical effectors or epigenetic remodeling, may selectively amplify WNT pathway output in this context. The molecular mechanisms for preferential enrichment of WNT, Notch, and Hedgehog gene signatures in K-Ras^G12C^ tumors warrants further investigation.

Our results highlight transcriptional changes associated with distinct K-Ras Gly12 alleles, however, such regulation is likely influenced not only by immediate signaling-mediated gene expression responses but also by underlying epigenetic mechanisms. Recent studies have demonstrated that individual K-Ras alleles can engage divergent epigenetic programs during pancreatic tumorigenesis, contributing to stable transcriptional reprogramming [49]. In addition, K-Ras driven DNA methylation has been shown to occur independent of canonical MAPK effector signaling in a cell-type dependent manner [50]. In light of these observations, it will be important to further investigate how allele-specific signaling shapes the epigenetic landscape and transcriptional plasticity in the context of colorectal tumor.

Taken together, our data demonstrate that different substitutions at the K-Ras codon 12 differentially modulate downstream signaling networks, leading to distinct phenotypic outcomes, including differences in tumor burden, survival, transcriptional programs, and therapeutic vulnerabilities. These findings advance our understanding of mutant-specific oncogenic signaling in the context of colon cancers and provide a framework for developing tailored therapeutic strategies for K-Ras mutant colorectal cancers.

## Supplementary information

Supplemental Figures

## Data Availability

The datasets generated for this study are publicly available in the NCBI Gene Expression Omnibus (accession number GSE301459). All other raw data are available, upon request, from the corresponding author.
