# Supplementary material for: The impact of K-Ras Gly12 mutants on homeostasis and tumorigenesis in the colonic epithelium
Source: Oncogene. 2026 Apr 10;45(19):1828–39. doi: 10.1038/s41388-026-03771-3 (PMC13139037; doi:10.1038/s41388-026-03771-3)
Supplement: Supplementary file 1 — Supplemental Figures [file 41388_2026_3771_MOESM1_ESM.pdf]

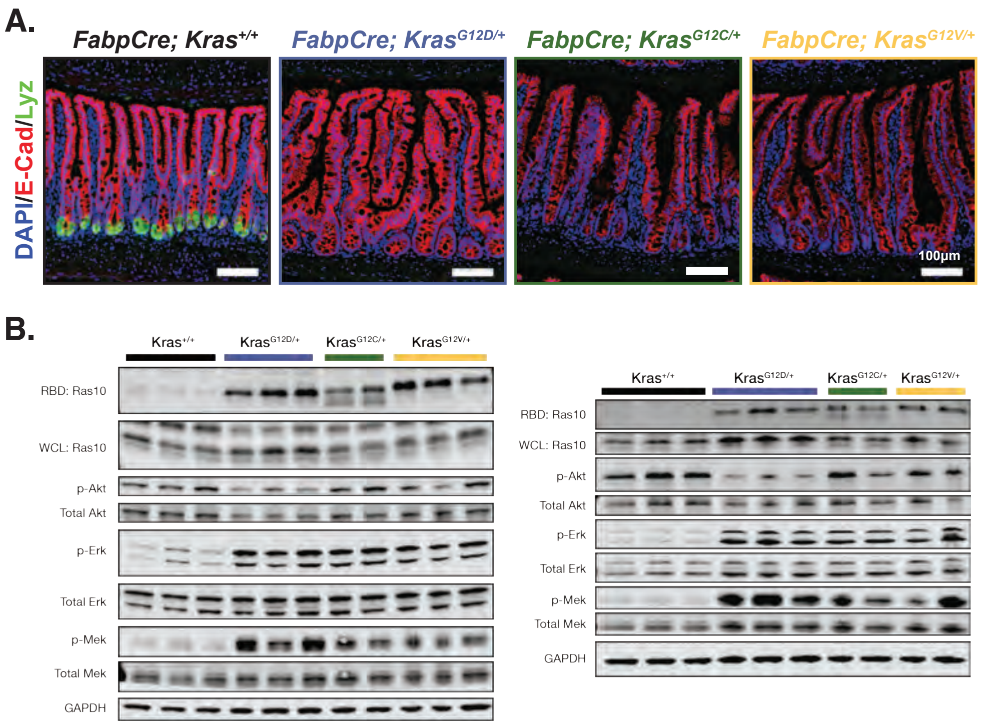

Figure S1. Analysis of the effect of K-Ras Gly12 mutants on the intestinal epithelium. (A) Assessment of secretory cell lineages in small intestinal epithelium expressing different Kras allele using immunofluorescence staining for Paneth cells. Representative immunofluorescence images of the ileum from Fabp1-Cre mice with the indicated Kras genotypes, displaying for E-Cadherin (Magenta), a marker for epithelial cells, and lysozyme, a maker for Paneth cells. Scale bars indicate 100μm. All three Kras Gly12 mutants completely suppresses the secretory cell lineage differentiation. (B) Western blot analysis for Ras activation and its downstream signaling components in colons carrying the indicated Kras alleles. Each lane contains lysate from an individual mouse.

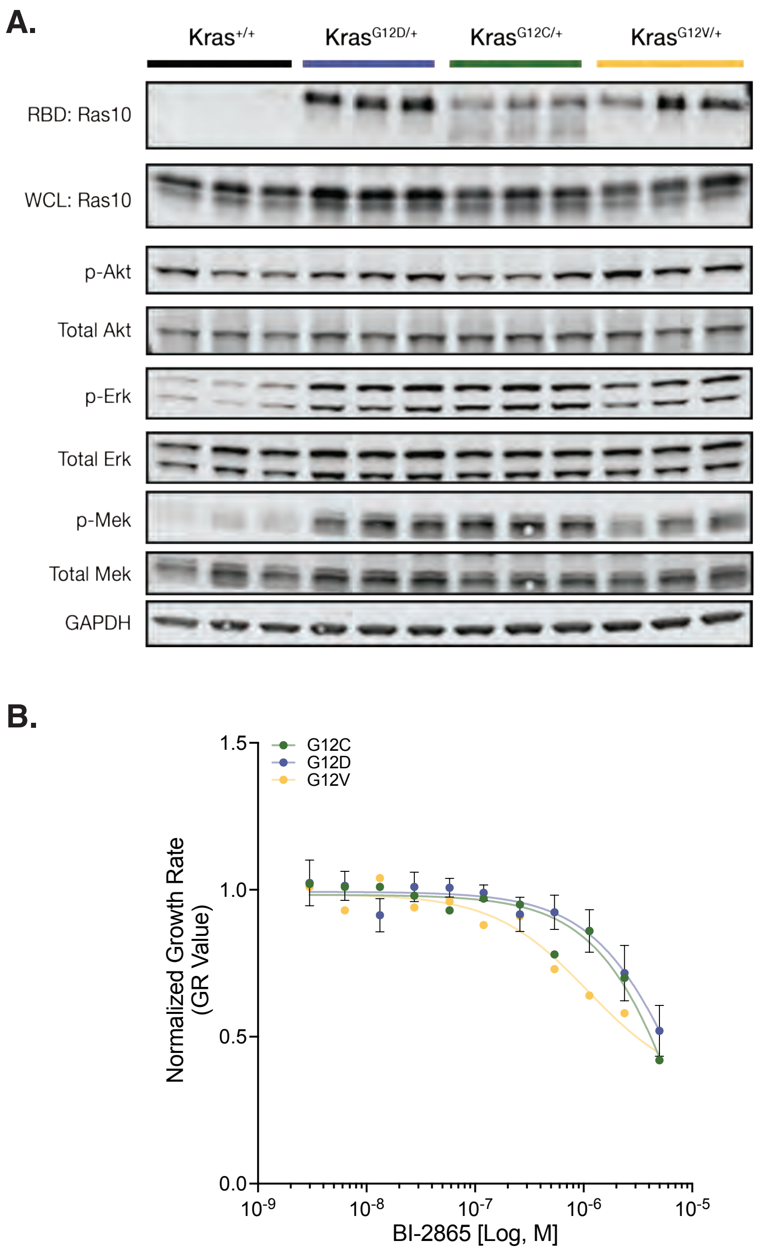

Figure S2. Analysis of the effect of K-Ras Gly12 mutants on the colon tumor. (A) Representative western blotting analysis for Ras activation and its downstream signaling components in colon tumors carrying the indicated Kras alleles. Each lane contains lysate from an individual mouse. (B) Response of colon tumor organoids carrying different Kras Gly12 mutant alleles to BI-2865, a pan KRAS inhibitor which targets to GDP-bound form of KRAS. Intriguingly, K-Ras^{G12V} organoids are more sensitive to BI-2865, whereas those expressing K-Ras^{G12C} or K-Ras^{G12D} exhibit similar response. For this experiment, EGF was included in the culture conditions, and three independent clones were used for K-Ras^{G12D} and K-Ras^{G12C}, and one clone was used for K-Ras^{G12V}. Each dot in inhibitor response curve represents the average of triplicates of each clone from same genotype across two independent experiments, with curve fitting using nonlinear regression.

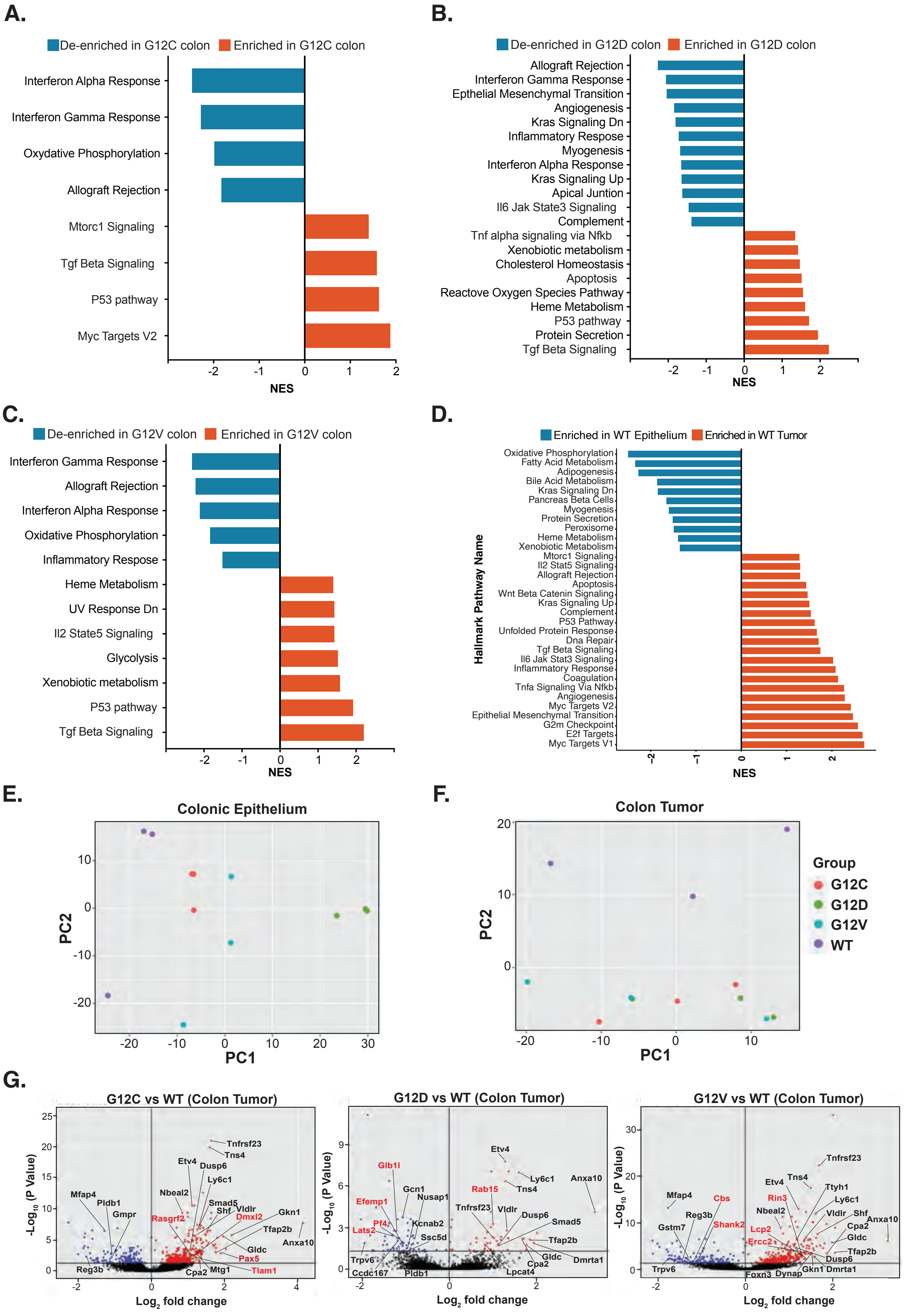

A.
De-enriched in G12C colon
Enriched in G12C colon
Interferon Alpha Response
Interferon Gamma Response
Oxydative Phosphorylation
Allograft Rejection
Mtorc1 Signaling
Tgf Beta Signaling
P53 pathway
Myc Targets V2
NES
B.
De-enriched in G12D colon
Enriched in G12D colon
Allograft Rejection
Interferon Gamma Response
Epthelial Mesenchymal Transition
Angiogenesis
Kras Signaling Dn
Inflammatory Respose
Myogenesis
Interferon Alpha Response
Kras Signaling Up
Apical Juntion
Il6 Jak State3 Signaling
Complement
Tnf alpha signaling via Nfkb
Xenobiotic metabolism
Cholesterol Homeostasis
Apoptosis
Reactove Oxygen Species Pathway
Heme Metabolism
P53 pathway
Protein Secretion
Tgf Beta Signaling
NES
C.
De-enriched in G12V colon
Enriched in G12V colon
Interferon Gamma Response
Allograft Rejection
Interferon Alpha Response
Oxidative Phosphorylation
Inflammatory Respose
Heme Metabolism
UV Response Dn
Il2 State5 Signaling
Glycolysis
Xenobiotic metabolism
P53 pathway
Tgf Beta Signaling
NES
D.
Enriched in WT Epithelium
Enriched in WT Tumor
Hallmark Pathway Name
Oxidative Phosphorylation
Fatty Acid Metabolism
Adipogenesis
Bile Acid Metabolism
Kras Signaling Dn
Pancreas Beta Cells
Myogenesis
Protein Secretion
Peroxisome
Heme Metabolism
Xenobiotic Metabolism
Mtorc1 Signaling
Il2 Stat5 Signaling
Allograft Rejection
Apoptosis
Wnt Beta Catenin Signaling
Kras Signaling Up
Complement
P53 Pathway
Unfolded Protein Response
Dna Repair
Tgf Beta Signaling
Il6 Jak Stat3 Signaling
Inflammatory Response
Coagulation
Tnfa Signaling Via Nfkb
Angiogenesis
Myc Targets V2
Epithelial Mesenchymal Transition
G2m Checkpoint
E2f Targets
Myc Targets V1
NES
E.
Colonic Epithelium
PC1
PC2
F.
Colon Tumor
PC1
PC2
Group
G12C
G12D
G12V
WT
G.
G12C vs WT (Colon Tumor)
G12D vs WT (Colon Tumor)
G12V vs WT (Colon Tumor)
-Log10 (P Value)
Log2 fold change
Tnfrsf23
Tns4
Etv4
Dusp6
Ly6c1
Mfap4
Pldb1
Gmpr
Nbeal2
Smad5
Shf
Vldlr
Rasgrf2
Dmxl2
Gkn1
Tfap2b
Anxa10
Gldc
Pax5
Tlam1
Reg3b
Cpa2
Mtg1
Glb1l
Gcn1
Nusap1
Efemp1
Pf4
Lats2
Kcnab2
Ssc5d
Trpv6
Ccdc167
Pldb1
Rab15
Tnfrsf23
Vldlr
Dusp6
Smad5
Tfap2b
Gldc
Cpa2
Lpcat4
Dmrta1
Cbs
Reg3b
Gstm7
Shank2
Rin3
Nbeal2
Lcp2
Ercc2
Ttyh1
Foxn3
Dynap
Gkn1
Dmrta1

Figure S3. Analysis of dysregulated pathways in colonic epithelium harboring Kras Gly12 mutant alleles and in Kras WT colon tumor. (A-C) GESA analysis for analyzing altered signaling pathways in each Kras Gly12 mutant compared to Kras WT in colon epithelium. (D) GESA analysis for determining signaling pathways alteration in Kras WT tumor compared to Kras WT colonic epithelium. (E-F) Principal component analysis of mouse colonic epithelium (E) and colon tumors samples (F) expressing different K-Ras mutants. In colonic epithelium, a clear separation is observed between different genotypes, whereas no distinct separation is evident between colon tumors expressing K-Ras Gly12 mutants. (G) Volcano plots display DEGs in colon tumors expressing K-RasG12C (left) or K-RasG12D (middle) or K-RasG12V (right) compared to those expressing K-Ras WT. Genes labeled in black represent agnostic genes commonly regulated across all three K-Ras Gly 12 mutants, whereas genes labeled in red denote allele-specific DEGs.

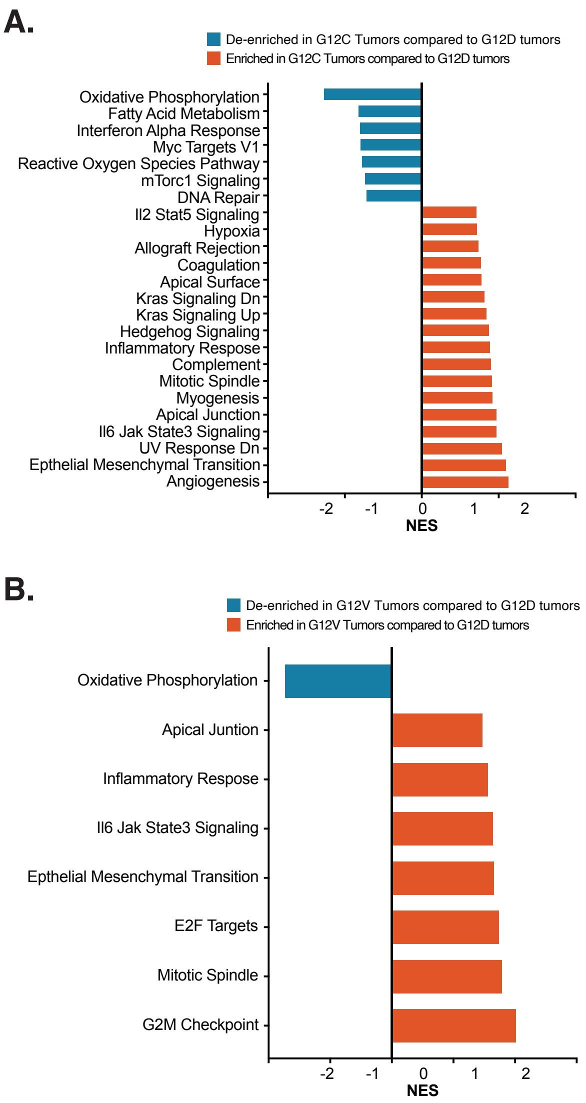

Figure S4. Analysis of dysregulated pathways in colonic tumors harboring Kras Gly12 mutant alleles. GESA analysis for determining signaling pathways alteration in colon tumors carrying Kras^{G12C} (A) or Kras^{G12V} (B) compared to those with Kras^{G12D}.

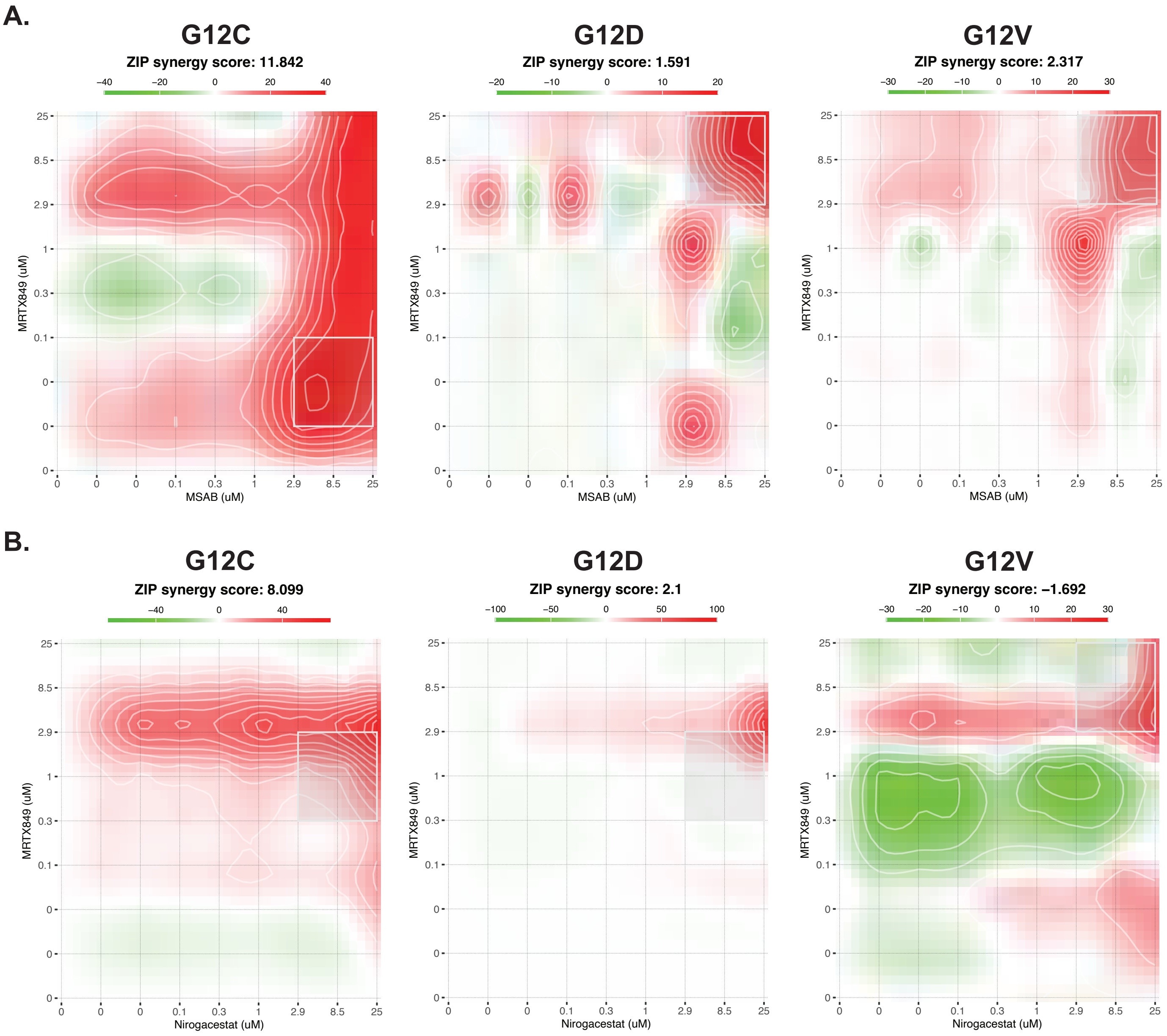

Figure S5. Combination treatment analysis of MRTX849 with Wnt or Notch pathway inhibitors in tumor organoids harboring Kras Gly12 mutants. (A) ZIP Synergy contour plots showing the combination effect between MRTX849 and MSAB in tumor organoids expressing K-Ras^{G12C}, K-Ras^{G12D} or K-Ras^{G12V}. Red regions indicate synergistic interaction, green regions indicate antagonistic effect, and white rectangles highlight concentration ranges exhibiting the most synergistic effect. Among the three genotypes, K-RasG12C organoids displayed the highest synergy scores, indicating a strong synergistic effect between KRAS inhibition and Wnt/ -catenin pathway blockade, whereas K-Ras^{G12D} and K-Ras^{G12V} organoids showed minimal or modest effect. (B) Synergy contour plots illustrating the combination effect between MRTX849 and Nirogacestat in tumor organoids expressing K-Ras^{G12C}, K-Ras^{G12D} or K-Ras^{G12V}. K-Ras^{G12C} organoids demonstrated the strongest addictive effect, while K-Ras^{G12D} organoids showed minimal response and K-Ras^{G12V} organoids exhibited an overall antagonistic profile. For all plots, synergy scores were calculated using the ZIP model, and the plots shown represent a single representative result from three independent experiments.

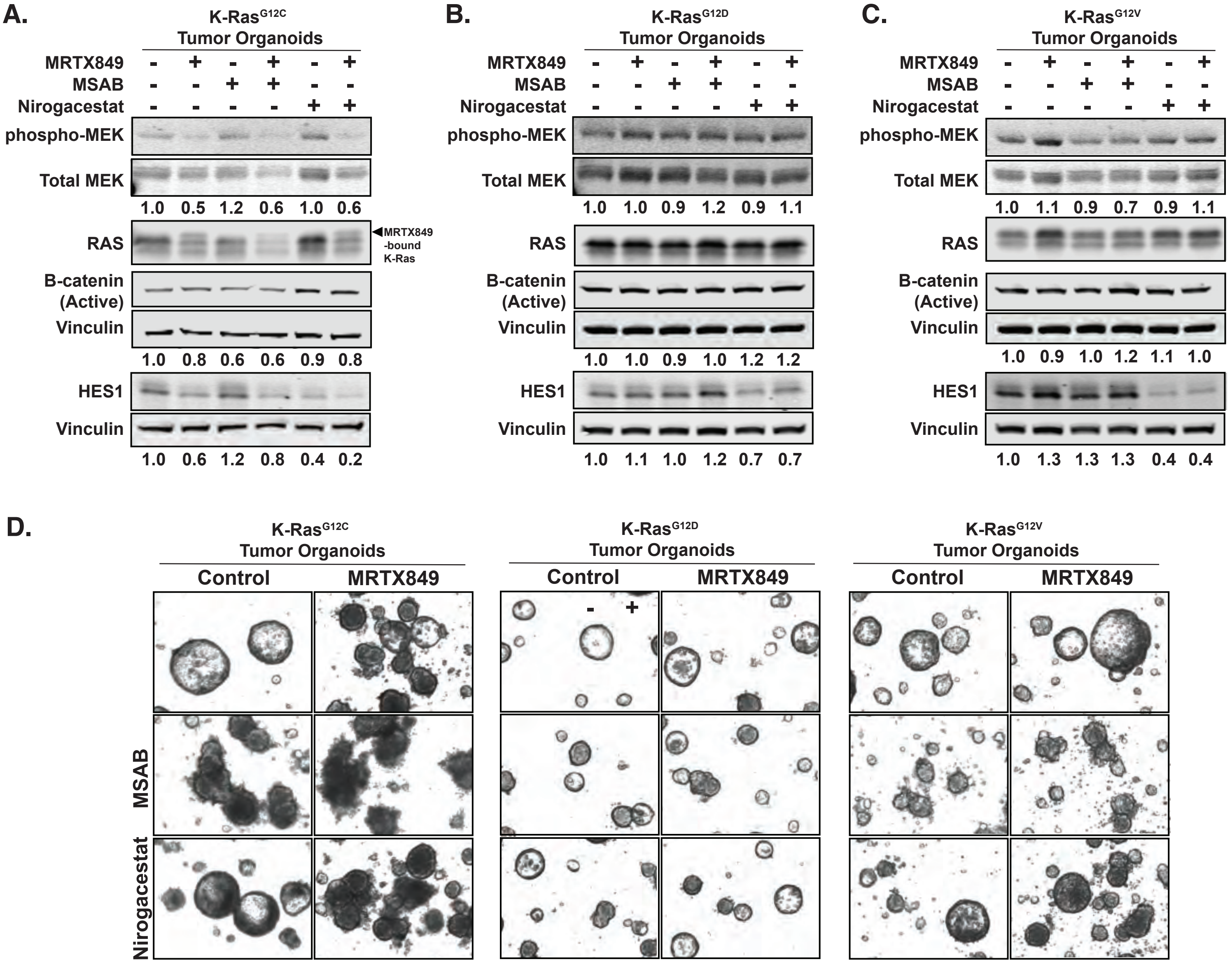

Figure S6. Analysis of canonical downstream signaling pathways following inhibitor treatments in K-Ras Gly12 mutant tumor organoids. (A-C) Western blotting analysis of canonical signaling pathway 24 hours after treatment with 2 μM MRTX849 alone or in combination with either 10 μM MSAB or 5μM Nirogacestat in tumor organoids expressing K-Ras^{G12C} (A). K-Ras^{G12D} (B), or K-Ras^{G12V} (C). Phospho-MEK and total MEK were analyzed to assess MAPK pathway activity. As expected, HES1, a transcriptional target of Notch signaling pathway was reduced upon Nirogacestat treatment, confirming effective Notch blockade. In K-Ras^{G12C} tumor organoids, MRTX849 treatment induced a characteristic mobility shift in K-Ras band, consistent with compound binding, whereas no such shift was observed in K-Ras^{G12D} or K-Ras^{G12V} organoids. Interestingly, the level of non-phosphorylated β-catenin was selectively decreased by MSAB treatment only in K-RasG12C tumor organoids at same concentration, suggesting allele-dependent sensitivity to β-catenin inhibition. (D) Representative bright-field images of K-Ras Gly12 tumor organoids treated for 24 hours with 2 μM MRTX849 alone or in combination with either 10 μM MSAB or 5 μM Nirogacestat. Images were captured immediately before sample harvest for western blot analysis shown in panels (A-C). In K-Ras^{G12C} organoids (left panels), inhibitor treatment, particularly the combination of MRTX849 with MSAB or Nirogacestat, induced pronounced morphological deterioration characterized by loss of structural integrity, surface disintegration, and collapse of the spherical architecture consistent with impaired viability. In contrast, K-Ras^{G12D} organoids (middle panels) showed minimal morphological alterations under the same condition, while K-Ras^{G12V} organoids (right panels) showed moderate structural changes, including reduced size and partial lumen collapse upon inhibitor exposure, with a mild compaction phenotype specifically observed after MSAB treatment, indicating a weaker yet detectable morphological response.
